# Compliance with Multiple Health Behaviour Recommendations: A Cross-Sectional Comparison between Female Cancer Survivors and Those with no Cancer History

**DOI:** 10.3390/ijerph16081345

**Published:** 2019-04-15

**Authors:** Daniel N Tollosa, Meredith Tavener, Alexis Hure, Erica L James

**Affiliations:** 1 School of Medicine and Public Health, University of Newcastle, Callaghan, Newcastle, NSW 2308, Australia; meredith.tavener@newcastle.edu.au (M.T.); alexis.hure@newcastle.edu.au (A.H.); erica.james@newcastle.edu.au (E.L.J.); 2 College of Health Sciences, Mekelle University, Mekelle 046, Ethiopia; 3 Hunter Medical Research Institute, Kookaburra Circuit, New Lambton Heights, Newcastle, NSW 2305, Australia

**Keywords:** multiple health behaviours, adherence, cancer survivors, ALSWH

## Abstract

Lifestyle behaviours have an important role in preventing cancer, reducing treatment side effects, and improving survival and quality of life for cancer survivors. This study investigated adherence to multiple lifestyle behaviours among women with and without a cancer history. From the Australian Longitudinal Study on Women’s Health (ALSWH) surveys, 2407 cancer survivors and 3896 controls (cancer free population) were identified. Based on the World Cancer Research Fund/American Institute for Cancer Research (WCRF/AICR) recommendations, adherence to six health behaviours (smoking, physical activity, fruit and vegetable intake, alcohol consumption, sugary drink intake, and Body Mass Index [BMI]) were assessed. Overall adherence was low, and there were no differences between survivors and controls on adherence to any of the six individual health behaviours. However, both recent and long-term cancer survivors were more likely than controls to adhere to multiple health behaviours (*p* < 0.05). When participants with melanoma or non-melanoma skin cancer were excluded, adherence was less likely (but not significant) in the cancer group than controls. Higher education (*p* < 0.01), being married (*p* < 0.01), and lower comorbidity of chronic illnesses (*p* < 0.01) were significantly associated with adherence to multiple lifestyle behaviours. Overall, the findings suggest that a cancer diagnosis may result in increased compliance with multiple health behaviour guidelines.

## 1. Introduction

The population of cancer survivors is growing. In 2012 alone, 32.6 million people were living with a cancer diagnosis worldwide [1]. Whilst increasing survival rates from cancer is encouraging, poor quality of life and cancer recurrences are major challenges for this population [1,2]. Healthy lifestyle habits, such as avoiding tobacco, engaging in physical activity, a healthy diet, and maintaining a healthy body weight have an important role both for primary cancer prevention of a second cancer, and also in reducing treatment side effects and improving the effectiveness of cancer treatment [1,3,4]. 

It is strongly advised that cancer survivors be holistic, and adopt health behaviour recommendations as a lifestyle package, rather than making changes to just one behaviour [1,5]. The potential additive effect of health behaviours (e.g., physical activity plus a healthy diet to maintain a healthy body weight) could produce a synergistic effect greater than each behaviour produces independently [6]. Hence, the need for behavioural change interventions targeting multiple health behaviours [7,8].

Whilst evidence is limited and inconsistent, there are a number of studies suggesting that cancer survivors make positive changes to health behaviours after a cancer diagnosis [9,10,11,12], and cancer survivors have healthier lifestyle behaviours than those who are cancer-free [13,14,15]. These findings emanate the notion that the period after a cancer diagnosis be considered as a “teachable moment”, whereby cancer survivors may be receptive to lifestyle change messages and may be more motivated to make healthy behaviour changes [16,17].

Differences may exist in health behaviours by duration of cancer survivorship (long-term versus recent survivors). In a U.S. prospective cohort study, the Prostate, Lung, Colorectal, and Ovarian (PLCO) Cancer Screening Trial revealed that recent cancer survivors were more likely to engage in moderate and strenuous physical activity when compared to long-term survivors, but there was no difference for BMI or smoking status [13]. This might suggest changes in health behaviour are not stable across the cancer continuum. 

Although there are studies that have investigated individual health behaviours of cancer survivors [6,18,19,20], and some studies that have compared these behaviours to a cancer-free population [13,14,15], evidence is limited regarding the concurrent practice of healthy lifestyle behaviours of cancer survivors. Therefore, the current study sought to examine (i) adherence with multiple health behaviour recommendations among recent and long-term cancer survivors (with and without melanoma and non-melanoma skin cancer) compared to a cancer-free population, and (ii) to identify factors associated with high adherence to multiple health behaviour recommendations. 

## 2. Materials and Methods 

### 2.1. Data Source

Data for this study are from the 1946–1951 cohort (aged 45–50 years at baseline in 1996) of the Australian Longitudinal Study on Women’s Health (ALSWH). ALSWH is a longitudinal population-based survey conducted every three years. For more details on the ALSWH survey, visit www.alswh.org.au. Briefly, over 40,000 women responded to a baseline survey sent out in 1996 to three different cohorts: women born between 1921–1926 (aged 70–75 years), women born between 1946–1951 (aged 45–50 years), and women born between 1973–1978 (aged 18–23 years) [21]. In 2012/2013, a new cohort of young women born between 1989–1995 (aged 18–23 years) was also recruited [22]. The survey takes a comprehensive view of different aspects of women’s health and health-related issues, including lifestyle behaviours, such as diet, physical activity, smoking, and alcohol intake. For the current study, the dependent variable (lifestyle behaviours) and covariates are taken from the eighth ALSWH survey of the 1946–1951 cohort, collected in 2016, when the women were aged 63–70 years.

### 2.2. Study Participants

The ALSWH survey asked women a series of questions related to diagnosis of chronic diseases, including various cancer types (breast, bowel, cervical, lung, skin (including melanoma), and “other cancer”). Those women who answered “yes” to the following survey question were identified as cancer survivors: “In the past three years, have you been diagnosed with or treated for breast, lung, cervical, bowel (colorectal), skin (melanoma and non-melanoma), or ‘other cancer’?”

The ALSWH item that assesses cancer diagnosis does not distinguish between melanoma and non-melanoma skin cancer, so subsequently these cancers were combined for the analysis. However, there is potential that women diagnosed with a non-melanoma skin cancer may experience a very different treatment journey (when compared to other cancer types with more intensive treatment and disease-related side effects). For the purpose of this study, we report differences in compliance with multiple health behaviour recommendations for the whole cancer group, and for the cancer group once all skin cancer participants are removed.

Cancer survivors identified from the 2013 ALSWH survey were classified as long-term survivors (3-to-6 years survival time) and from the 2016 survey as recent survivors (<3 year survival time). For comparison, a cancer free population was identified within ALSWH—those women who had never reported a cancer diagnosis in any ALSWH surveys since the baseline survey in 1996. From data linked to cancer registries, sensitivity and specificity of self-reposted cancer diagnosis is high in ALSWH surveys [23].

### 2.3. Measures

#### Covariates

Socio-economic and demographic variables, including age (ranged from 63 to 70 years), marital status (married, not married), occupation (paid, unpaid job), residential state, and geographic location of residence (urban, not urban) were retrieved from the 2016 survey. With the assumption educational status (no formal education, certificate, and university degree) was not likely to have significantly changed since the women were 45–50 years, we used the baseline characteristics in the current study.

The presence of co-morbidities was assessed with the question: “In the past three years, have you been diagnosed with or treated for the following chronic illness….?” In the current study, we included nine conditions: diabetes, osteoarthritis, osteoporosis, heart disease, hypertension, stroke, asthma, depression, and anxiety.

### 2.4. Health Behaviours

#### 2.4.1. Smoking

Respondents were asked about smoking behaviour and classified into “do not smoke” (respondents who did not smoke any tobacco product currently) or “currently smokes” (smokes cigarettes either daily, weekly, or less than weekly). The survey also identified a history of smoking (those who had smoked cigarettes previously but not currently).

#### 2.4.2. Physical Activity

Physical activity was assessed via the Active Australia survey, which is validated for use with older populations (age ≥ 65 years) [24]. ALSWH participants recorded the total weekly hours of activities (in 10 min bouts) in the past week: light intensity activities (e.g., walking briskly), moderate leisure activiimming, dancing), and vigorous leisure activities ty (e.g., tennis, moderate exercise classes, recreational sw(e.g., competitive sport, vigorous cycling, running, swimming). Total Physical Activity (TPA) score was calculated using Metabolic Equivalent Task (MET), i.e., computed by multiplying the sum weekly physical activities (in minutes) by the assigned MET value (on average), i.e., light intensity = 3.3 METs, moderate-intensity = 4 METs, and vigorous leisure activities = 7.5 METs. The MET values are adopted from the compendium of physical activities [25]. 

International organizations, such as the World Health Organization (WHO), American Cancer Society (ACS), and the World Cancer Research Fund/American Institute for Cancer Research (WCRF/AICR), recommend that cancer survivors should undertake at least 600 MET-minutes/week of physical activity, which is equivalent to 150 min each week of brisk walking or 75 min per week of running. To measure compliance with the recommendation and to be comparable with other studies, four ordered physical activity levels were created: level 1 (sedentary) = 0 −< 40 MET min/week, level 2 (insufficiently active) = 40 −< 600 MET-min/week), level 3 (sufficiently active) = 600 −<1200 MET-min/week, and level 4 (very active) ≥1200 MET-min/week. 

#### 2.4.3. Fruit and Vegetable Intake

Respondents were asked how many pieces of fresh fruit they consumed per day (1 piece of fruit refers to ½ cup of diced fruit, berries, or grapes); responses ranged from “Don’t eat fruit” to “4 or more pieces of fruit per day”. They were also asked how many serves of vegetables they consumed per day (a serve refers to 1/2 cup of cooked vegetables or a cup of salad vegetables). Responses ranged from “Don’t eat vegetables” to “5 or more serves of vegetables per day”.

Responses were used to determine the daily fruit consumption (categorized into <2 and ≥2 serves of fruit per day) and vegetable consumption (categorized into <5 and ≥5 serves of vegetables). For analysis, and to be in line with the recommendations, those respondents who consumed more than two pieces of fruit and five serves of vegetables were grouped together to determine adherence to combined fruit and vegetable behaviour.

#### 2.4.4. Alcohol Consumption

Participants reported how often they usually drink alcohol. Eight options were provided: “I have never drunk alcohol in my life”, “I never drink alcohol but I have in the past”, “I drink rarely”, “Less than once a week”, “On 1 or 2 days a week”, “On 3 or 4 days a week”, “On 5 or 6 days a week”, and “Every day”. Those who responded in the affirmative to drinking alcohol currently were further asked about the number of standard drinks they usually have: “1 or 2 drinks per day”, “3 or 4 drinks per day”, “5 to 8 drinks per day”, and “9 or more drinks per day”. Although the recommendation of alcohol intake for cancer survivors (women) is limited to less than one standard drink per day, the ALSWH data does not allow categorization based on this recommendation. Thus, a category of less than two standard drinks was used for the current study.

#### 2.4.5. Body Mass Index (BMI)

Women reported their current weight and height (“How much do you weigh?” and “How tall are you without shoes?”), from which BMI was calculated and categorized into underweight (BMI of <18.5 kg/m^2^), a healthy or normal weight (BMI of 18.5–24.9 kg/m^2^), overweight (BMI of 25.0–29.9 kg/m^2^), and obese (BMI of 30.0 kg/m^2^ or more). 

#### 2.4.6. Sugary Drinks

Women reported their consumption of non-diet soft drinks (e.g., Coke) over the last 12 months. Ten options are given for this question; “Never”, “1 to 3 times per month”, “1 time per week”, “2 times per week”, “3 to 4 times per week”, “5 to 6 times per week”, “1 time per day”, “2 times per day”, and “3 or more times per day”. Avoiding sugary drinks is recommended for cancer survivors; thus, participants were categorized into two groups: “never drink” and “those who consumed at least once per month”. 

#### 2.4.7. Multiple Health Behaviours

“Multiple health behaviours” was determined by summative index scoring method, which gives equal weight for each behaviour. First, adherence to each health behaviour (smoking, physical activity, alcohol, BMI, fruit and vegetables, and sugar drinks) was determined based on the WCRF/AICR recommendations (Table 1) [1], in which 1 point was given for those who met the recommendation; 0 (zero) points for those who did not. The individual health behaviour score was then summed to produce a multiple health behaviours score, ranging from zero (i.e., adherence to none of the recommendations) to six (i.e., adherence to all health behaviour recommendations). The higher number indicates greater adherence to recommended behaviours. Considering other similar studies [18,19,26], the multiple health behaviours score was categorized into less than or equal to one (<1), 2–3, 4, and 5–6 behaviours.

### 2.5. Data Analysis

An explanatory analysis was undertaken to understand the distribution and summarize the main characteristics of the study variables. Chi2 test was used to test the difference in adherence to heath behaviours between controls (cancer-free population) and cancer survivors. 

Ordinal (proportional odds) regression model was used to estimate the odds for adherence to the concurrent practice of healthy behaviours (order from ≤1 behaviour, 2–3 behaviours, 4 behaviours, and 5–6 behaviours) by cancer survivorship, which is adjusted for covariates (age group, reported comorbid chronic diseases, residential area, education status, and occupation status). 

Skin cancer is often associated with exposure to Ultraviolet (UV) radiation, and evidence is limited on the other health behaviours, including physical activity, smoking, and dietary factors; however, skin cancer survivors are at higher risk of developing other cancer types or second primary cancers [27]. Thus, the health behaviours included in this study are also important and recommended for them. 

Each model was adjusted for the variables that showed a significant or close-to-significant (*p* ≤ 0.2) association in the bivariate analysis. The cancer-free population was used as a reference group for regression analysis. The result was considered significant at a 95% level of significance (*p* < 0.05). A weighted sample analysis was used, since ALSWH participants from the rural areas of Australia were intentionally oversampled. Proportional odds assumption or the parallel regression assumption was evaluated by Brant’s test (*p* > 0.05).

## 3. Results

**Figure 1:**
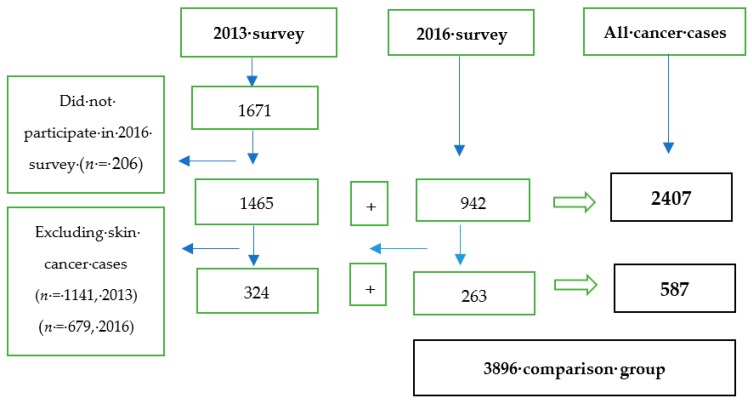
Selection of the study participants.

A total of 2613 women reported a diagnosis of cancer (all types) in the 2013 (*n* = 1671, long-term survivors) and 2016 (*n* = 942, recent survivors) ALSWH surveys. Of women who reported cancer in the 2013 survey, 206 did not participate in the subsequent 2016 survey. For this reason, the total number of participants used for the current study was 2407 (1465 from 2013 survey as long-term survivors and 942 from the 2016 survey as recent survivors). Excluding skin cancer survivors reduces the total number of participants to 587, i.e., 324 long-term survivors and 263 recent survivors. From all participants, the majority were skin cancer survivors (74%), followed by breast cancer survivors (13.4%). For comparison, 3896 women who replied to the 2016 survey, with no history of a cancer diagnosis, were identified as the cancer free-population (Figure 1).

### 3.1. Characteristics of Cancer Survivors and Controls

There were significant differences between cancer survivors (including skin cancer) and the control group (cancer-free population) across a range of demographic variables, but the difference became non-significant for most of the characteristics when excluding skin cancer survivors. Significant differences were not found between recent and long-term survivors in most of the demographic and health characteristics, except for baseline educational status among survivors, including skin cancer (*p* < 0.038), and occupational status among survivors, excluding skin cancer (*p* = 0.048) (Table 2).

### 3.2. Adherence to Individual Health Behaviours 

Overall, there was no significant difference between cancer survivors (with and without skin cancer survivors) and controls for individual health behaviours, except on adherence to sugary drink intake, whereby cancer survivors (excluding skin cancer survivors) were less likely to adhere to sugary drink recommendation (AOR: 0.68, 95% CI; 0.53, 0.87) by 32% compared to the cancer free group (Table 3 and Table 4). 

In this sample, long-term cancer survivors’ adherence to individual health behaviours did not significantly differ from the cancer-free population. Recent cancer survivors (with skin cancer) were 25% more likely to adhere to BMI of 18.5–24.9 kg/m^2^ (AOR: 1.25, 95% CI: 1.01, 1.51) and 20% less likely to adhere to >2 pieces of fruit intake per day (AOR: 0.80, 95% CI: 0.66, 0.97) than those in the cancer-free population, respectively (Table 4).

### 3.3. Adherence to Multiple Health Behaviours and Associated Factors

Of the six behaviours assessed in this study, the mean adherence was 3.55 (SD: 1.12) for controls, 3.59 (SD: 1.10) for survivors with skin cancer, and 3.42 (SD: 1.08) for survivors without skin cancer. The majority of cancer survivors, 42.5% (with skin cancer) and 50.4% (without skin cancer), adhered to 2–3 healthy lifestyle behaviours. Adherence to 5–6 health behaviours was considerably low, only 21.8% (with skin cancer) and 16% (without skin cancer) (Table 3). 

Compared to the cancer free population, we found that adherence to multiple health behaviours was significantly different in recent and long-term survivors (with skin cancer); however, the difference became non-significant for survivors without skin cancer (Table 5). The odds for recent and long-term survivors (with skin cancer) to achieve a higher category of adherence to multiple behaviours were 19% (AOR: 1.19, 95% CI: 1.04, 1.37) and 14% (AOR: 1.14, 95% CI: 1.02, 1.29) higher than the control group, respectively. 

For all survivors, being older (69–73 years), living in urban area, being married, and having a higher level of education significantly improved the adherence to multiple health behaviours. The effect of education and marital status remained significant for survivors, excluding skin cancer. 

This study also revealed that comorbid chronic illnesses in cancer survivors significantly impede adherence to multiple health behaviours. Nearly 70% of survivors reported at least one comorbid chronic illness. Compared to survivors who did not report other chronic diseases, those survivors (with skin cancer) who reported 1–2 and >3 chronic diseases were less likely to be in the higher category of adherence to multiple behaviours by 34% (AOR: 0.66, 95% CI: 0.59, 0.73) and 64% (AOR: 0.36, 95% CI: 0.30, 0.41), respectively. Similarly, reporting 1–2 and >3 chronic diseases reduced the adherence to be in the higher category of multiple behaviours by 36% (AOR: 0.64, 95% CI: 0.57, 0.73) and 64% (AOR: 0.36, 95% CI: 0.30, 0.44) for survivors, excluding skin cancer (Table 5).

## 4. Discussion

Healthy lifestyle behaviours play an important role in improving the quality of life and longevity for cancer survivors [6]. This cross-sectional study has focused on examining whether or not a cancer diagnosis was associated with cancer survivors’ adherence to multiple lifestyle behaviours and identifying socio-demographic and health determinates. Overall, the study revealed that there was a considerable difference between all cancer survivors and the cancer-free population on compliance with multiple health behaviours, but the evidence was not sufficient for individual health behaviours and for survivors, excluding skin cancer.

Although the diagnosis of cancer has been suggested as a “teachable moment”, whereby survivors are motivated for health behaviour changes after a cancer diagnosis [16,17], research findings to date are largely inconsistent. Concerning adherence to individual lifestyle behaviours, the study revealed that there were no significant differences between cancer survivors and cancer-free population. These findings are similar to previous studies that compared individual health behaviours of cancer survivors with cancer-free populations [28,29,30].

A single health behaviour could be inadequate to improve overall survival and quality of life of cancer survivors [1,5]. Health behaviour interventions, therefore, need to be holistic and address multiple lifestyle factors, either sequentially or simultaneously [7,31]. However, few studies are available that measure cancer survivors’ concurrent health behaviour practices [18,19,26,32,33,34,35,36,37,38]. In the present study, around 22% (all cancer survivors) and 16% (survivors excluding skin cancer) adhered to at least 2/3 of the WCRF/AICR recommendations. This result was comparable to previous studies by Song, 2015 (20.6%) [35], and Smith, 2014 (27%) [34], but relatively lower compared to studies by Spector, 2015 (36.8%) [36], Inou-chio, 2013 (34.1%) [18] and Kanera, 2016 (40%) [19], and higher compared to studies by Winkles, 2016 (12%) [38] and Von-gruninegn, 2011 (18%) [37]. This discrepancy might be related to health policy and intervention focus on improving health behaviours in cancer survivor populations across countries; however, the overall adherence to multiple behaviours is very low, and thus integrated health promotion strategies that recognize the individual and social contexts are warranted.

Whilst a cancer diagnosis has an impact on cancer survivors’ behaviour changes, sustaining or maintaining the change is a major concern [39]. Park et al., 2015 [20] reported that time since cancer diagnosis was a strong determining factor for maintaining health behaviour change, in which long-term survivors were more likely relapse to health-risk behaviours (e.g., alcohol consumption and smoking) compared to recent survivors. Similarly, relative to the cancer free population, the current study suggests that the strength of adherence to a higher level of multiple behaviours was more noticeable in recent survivors compared to the long-term survivors. Excluding skin cancer survivors from the sample, however, provided a different result, in which the period following a diagnosis to cancer did not represent a "teachable moment” for health behaviour changes. This difference could be due to a smaller sample size for survivors excluding skin cancer, or perhaps skin cancer survivors demonstrate a different health behaviour compared to other cancer survivors. Further studies need to be done to clarify this ambiguity. Overall, empowering and continuous support of cancer survivors throughout the course of cancer survivorship is highly required. 

The co-occurrence and interaction between cancer and additional chronic illness could increase the burden of cancer and quality of life for many cancer survivors [40]. In the current study, seven out of ten survivors reported at least one chronic disease in addition to cancer, which related to metabolic, cardiovascular, mental health, and musculoskeletal illnesses. The prevalence was relatively low compared to the report by Eiken et al., 2007 (86%) [26], but significantly higher than a study in the USA (40%) [37]. A systematic review by Lee, et al., 2011 also reported a wide range; 0.4–90% prevalence of comorbidity among cancer survivors [38]. This discrepancy might be related to the survivor’s population and definition of comorbidity in the studies. In the present study, we found that the number of reported chronic diseases other than cancer significantly obstructed adherence to multiple behaviours in cancer survivors with and without skin cancer, thus there is a need to consider the complex interaction between cancer and other comorbidities in cancer survivorship care plans. 

This study had a number of limitations that need to be considered. Utilization of the ALSWH data for this analysis does introduce some potential limitations. First, the self-reported nature of the data collection in ALSWH survey did not objectively determine the participant health behaviour practice and cancer history, thus there could be under or over-reporting. Second, due to the three-year cycle of data collection used in the ALSWH study, there is the potential that some participants were diagnosed with and died from cancer in between survey cycles. Thus, there could be survivor bias, since only women who are still alive provide survey responses. This impacts the generalizability of our sample, as it potentially excludes women diagnosed with aggressive cancers, or those diagnosed with advanced disease. Moreover, it is not possible to classify survivors into recent and long-term using the common five-year cut off point. Third, the study is not representative for all gender groups, as only female cancer survivors participated. Fourth, questions related to alcohol consumption are not structured according to the WCRF/AICR recommendation, i.e., ≤1 SD per day for women; instead we used ≤2 SD per day. Fifth, ALSWH surveys do not distinguish melanoma and nonmelanoma skin cancer, thus they were analyzed together. 

In addition, the scoring approach used for adherence to multiple behaviours, i.e., creation of a summative index, implies each behaviour is equivalent. This issue has been raised by other authors exploring multiple health behaviours [8,41]. The alternative methods require continuous variables and result in reduced interpretability of change scores. Skin cancer survivors contribute significant numbers to the total sample size, thus excluding them could reduce the statistical power of detecting associations and comparability of the results with all cancer survivors group. All cancer types other than skin cancer were grouped together for analysis, and this could be a limitation, since the level of adherence to health behaviours could vary by cancer type. Although the available ALSWH data were comprehensive, we were unable to assess other important factors that could affect the adherence to health behaviours, such as cancer stage and cancer treatment received.

## 5. Conclusions

Overall adherence was low; however, both recent and long-term cancer survivors were more likely to adhere to health behaviours than controls, suggesting that a cancer diagnosis may result in increased compliance with multiple health behaviour guidelines. Level of education, marital status, and comorbidity of chronic illnesses were significantly associated with adherence to multiple lifestyle behaviours.

## Figures and Tables

**Table 1:** WCRF/AICR lifestyle recommendations for cancer survivors: operationalizing and scoring recommendations in the present study.

Variable	WCRF/AICR Recommendation	Measurement Scale	Operationalize	Scoring
Body weight	Be as lean as possible without becoming underweight and maintain body weight within the normal range	Body Mass Index (BMI)	BMI (kg/m^2^) < 18.5	0
			BMI = 18.5–24.9	1
			BMI = 25–29.9	0
			BMI ≥ 30	0
Physical activity	Avoid inactivity and return to normal daily activities as soon as possible following cancer diagnosis Aim to exercise at least 30 min per day or at least 150 min/week, which is equivalent to 600 MET/Week	Metabolic equivalent of task (MET) value	Level 1 (sedentary) = 0 −< 40 MET min/week	0
			Level 2 (insufficiently active) = 40 −< 600 MET min/week	0
			Level 3 (sufficiently active) = 600 −<1200 MET min/week	1
			Level 4 (very active) = ≥1200 MET min/week	1
Fruit intake	Consume at least 2 serves of fruit per day	Number of serves	≥2 serves of fruit per day	1
			<2 serves of fruit per day	0
Vegetable intake	Consume at least 5 portions/servings (>400 g) of a variety vegetables every day	Portion size (Serving)	≥5 serving of vegetable per day	1
			<5 serving of fruit and vegetable (F&V) per day	0
Smoking	Entirely avoided tobacco If you currently smoke or use tobacco in any form, ask your health professional about ways to quit	Yes/no	Current smoker	0
			Non-smoker or ex-smoker	1
Alcohol	Avoid alcoholic drinks, otherwise limit intake to two drinks for men and one drink for women a day.	Standard drink (1 SD refers to 10 grams of alcohol)	≤1–2 standard drink per day	1
			>2 standard drink per day	0
Sugary drinks	Avoid the intake of sugar drinks	Frequency	Not drink sugary drinks	1
			Drink at least once in a month	0

**Table 2:** Characteristics of cancer survivors (with and without skin cancer) and the control group.

Scio-Demographic and Health Characteristics	Controls (%)	Cancer Survivors Including Skin Cancer		P^a^	Cancer Survivors Excluding Skin Cancer		P^b^	All Cancer Cases (Long-Term and Recent Survivors)			
		Long-Term Freq., (%)	Recent Freq., (%)		Long-Term Freq., (%)	RecentFreq., (%)		With Skin Cancer (%)	P^c^	Excl. Skin Cancer (%)	P^d^
**Age**											
Total (Mean, SD)	3896 (66.9, 1.47)	1465 (67.2, 1.44)	942 (67.2, 1.43)	0.714	324 (67.2, 1.47)	263 (67.2, 1.43)	0.862	2407 (67.8, 1.42)	<0.01	587 (67.8, 1.42)	0.012
Age group											
63–67 years 68–70 years	60.8 39.2	57.0 43.0	56.9 43.1	0.529	57.1 42.9	58.2 41.8	0.658	53.4 46.6	<0.01	55.2 44.8	0.308
**Current residential area (N)**	3810	1436	919		320	256		2355		576	
Urban Not Urban	66.2 33.8	63.4 36.6	62.5 37.5	0.717	62.8 37.2	61.6 38.4	0.805	63.1 36.9	0.023	62.3 37.7	0.103
**Current marital status (N)**	3859	1456	932		322	259		2388		581	
Married Not married	66.2 33.8	64.7 35.3	66.3 33.6	0.515	63.8 36.2	62.2 37.8	0.742	65.3 34.7	0.582	63.1 36.9	0.249
**Current occupation (N)**	3767	1429	908		311	254		2,337		565	
Un paid job Paid job	76.1 23.9	79.4 20.6	82.6 17.4	0.129	76.4 23.6	84.8 15.2	0.049	80.7 19.3	<0.01	80.2 19.8	0.086
**Highest qualification (at baseline survey) (N)**	3863	1456	936		321	262		2597		660	
No formal education	13.6	10.8	12.4	0.038	13.6	15.9	0.274	11.4	0.015	14.7	0.699
Certificate (Intermediate/high school)	45.8	46.9	52.7		45.6	50.2		49.2		47.7	
Certificate (diploma/apprenticeship)	21.2	21.9	17.8		18.2	19.4		20.3		18.7	
University degree	19.4	20.4	17.1		22.6	14.5		19.1		18.9	
Reported comorbid chronic diseases * (N)	3896	1465	942		324	263		2407		587	
None One to two Three or more	36.3 51.6 12.1	30.7 51.1 18.2	29.1 53.8 17.1	0.602	32.2 51.3 16.5	31.3 52.9 15.7	0.952	30.1 52.2 17.7	<0.01	31.8 52.0 16.2	0.047

**P^a^:***p*-value for comparing long-term and recent survivors (with skin cancer); **P^b^:***p*-value for comparing long-term and recent cancer survivors (without skin cancer); **P^c^:***p*-value for comparing all cancer cases (including skin cancer) and controls (cancer-free population); **P^d^:***p*-value for comparing all cancer cases (excluding skin cancer) and controls; *include nine chronic diseases: diabetes, osteoarthritis, osteoporosis, heart disease, hypertension, stroke, asthma, depression, and anxiety.

**Table 3:** Descriptive statistics on adherence to individual and multiple lifestyle behaviours among cancer survivors (with and without skin cancer), compared with controls.

Lifestyle Behaviours	Controls (*n* = 4415)	Cancer Survivors Including Skin Cancer		P^a^	Cancer Survivors Excluding Skin Cancer		P^b^	All Cancer Cases (Long-Term and Recent Survivors)			
		Long-Term Freq., (%)	Recent Freq., (%)		Long-Term Freq., (%)	Recent Freq., (%)		Incl. Skin Cancer Survivors Freq., (%)	P^c^	Excl. Skin Cancer Survivors Freq., (%)	P^d^
Body Mass Index (BMI)											
<18.5 Kg/m^2^ 8.5–24.9 Kg/m^2^ 25 kg/m^2^–29.9 Kg/m^2^ ≥30 kg/m^2^ Met the recommendation Not met the recommendation	44 (1.2) 1254 (34.1) 1267 (34.5) 1111 (30.2)	19 (1.4) 485 (35.0) 461 (33.9) 434 (29.7)	17 (2.1) 317 (38.7 )281 (30.7) 286 (28.4)	*0.312*	4 (1.3) 86 (28.1) 97 (31.7) 119 (38.9)	6 (2.4) 89 (35.0) 71 (28.0) 88 (34.7)	*0.066*	36 (1.6) 802 (34.9) 742 (32.2) 720 (31.3)	0.530	10 (1.7) 175 (31.3) 168 (30.0) 207 (39.9)	0.130
	1254 (34.1) 2422 (65.9)	485 (34.9) 914 (65.1)	317 (38.7) 584 (61.3)	*0.156*	86 (28.1) 220 (71.9)	89 (35.0) 165 (65.0)	0.027 *	802 (34.8) 1498 (65.2)	0.796	175 (31.2) 385 (68.8)	0.544
Physical activity											
≤40 MET/week 40–600 MET /week 600–1200 MET/week ≥1200 MET/week Sufficient (≥600 MET/week) Insufficient (<600 MET/week)	752 (19.3) 888 (22.8) 754 (19.4) 1502 (38.5)	284 (18.6) 323 (22.1) 314 (21.2) 544 (38.1)	191 (17.7) 190 (21.1) 198 (19.7) 363 (41.5)	*0.633*	78 (24.1) 78 (24.1) 71 (21.9) 97 (29.9)	62 (23.5) 62 (23.5) 53 (20.2) 86 (32.7)	*0.856*	475 (19.7) 513 (21.3) 512 (21.3) 907 (37.7)	0.848	140 (23.8) 140 (23.8) 124 (21.1) 183 (31.2)	0.204
	2256 (57.9) 1640 (42.1)	858 (59.3) 607 (40.7)	561 (61.2) 381 (38.8)	*0.463*	168 (51.8) 156 (48.2)	139 (52.8) 124 (47.2)	*0.820*	1,419 (58.9) 988 (41.1)	0.685	307 (52.3) 280 (47.7)	*0.078*
Smoking											
Not smoke at all (met the recommendation)	3630 (94.6)	1381 (94.8)	888 (95.4)	*0.959*	302 (94.1)	243 (93.5)	*0.467*	2,269 (95.1)	0.246	545 (93.8)	0.311
Smoke; daily, weekly or monthly (not met the recommendation)	207 (5.4)	75 (5.2)	43 (4.6)		19 (5.9)	17 (6.5)		118 (4.9)		36 (6.2)	
Fruit consumption											
<2 pieces per day≥2 pieces per day	1356 (35.0) 2517 (65.0)	505 (34.6) 953 (65.4)	346 (37.0) 589 (63.0)	*0.181*	113 (35.0) 210 (65.0)	101 (38.5) 161 (61.5)	*0.250*	851 (35.6) 1542 (64.4)	0.261	214 (36.5) 371 (63.4)	0.231
Vegetable consumption											
<5 serving per day≥5 serving per day	3312 (85.6) 558 (14.4)	1236 (84.7) 223 (15.3)	800 (85.6) 135 (14.4)	*0.621*	284 (87.9) 39 (12.1)	223 (85.1) 39 (14.9)	*0.897*	2036 (85.1) 358 (14.9)	0.839	507 (86.6) 78 (13.3)	0.217
Fruit and vegetable											
<5 serving F&V per/day Not met both recommendations Met at least one recommendation ≥5 serving F&V per day	3460 (89.4) 1207 (34.9) 2253 (65.1) 409 (10.6)	1292 (88.6) 449 (30.8) 843 (57.8) 166 (11.4)	830 (88.7) 316 (33.8) 514 (55.0) 105 (11.3)	*0.569*	296 (91.6) 101 (34.1) 195 (65.9) 27 (8.4)	229 (87.4) 95 (41.5) 134 (58.5) 33 (12.6)	*0.582*	2,122 (88.7) 765 (36.1) 1357 (63.9) 271 (11.3)	0.935	525 (89.7) 196 (37.3) 329 (62.7) 60 (10.3)	0.360
Alcohol											
Never drink (current and ever) 1–2 SD per day >2 SD per day Met the recommendation Not met the recommendation	629 (16.7) 2710 (71.8) 434 (11.5)	249 (17.5) 1012 (71.0) 164 (11.5)	169 (18.5) 649 (71.1) 95 (10.4)	*0.237*	64 (20.5) 215 (68.7) 34 (10.9)	42 (16.5) 190 (74.5) 23 (9.1)	*0.733*	418 (17.9) 1661 (71.0) 259 (11.1)	0.699	106 (18.6) 405 (71.3) 57 (10.0)	0.701
	3339 (88.5) 434 (11.5)	1261 (88.5) 164 (11.5)	818 (89.6) 95 (10.4)	*0.402*	279 (89.1) 34 (10.9)	232 (90.9) 23 (9.1)	*0.480*	2079 (88.9) 259 (11.1)	0.427	511 (89.9) 57 (10.0)	0.719
Sugar drinks											
Never drink Drink at least once in a month	2947 (76.7) 896 (23.3)	1124 (77.3) 331 (22.7)	688 (74.3) 238 (25.7)	*0.083*	233 (72.2) 90 (27.8)	181 (69.1) 81 (30.9)	*0.380*	1812 (76.1) 569 (23.9)	0.197	414 (70.7) 171 (29.3)	0.001 *
Multiple behaviours											
≤1 behaviour 2–3 behaviours 4 behaviours 5–6 behaviours	134 (3.3) 1713 (44.0) 1254 (32.3) 795 (20.4)	41 (2.8) 619 (42.3) 484 (33.0) 321 (21.9)	28 (2.9) 405 (42.9) 305 (32.4) 204 (21.6)	*0.640*	10 (3.1) 165 (50.9) 103 (31.8) 46 (14.2)	8 (3.0) 131 (49.8) 76 (28.9) 48 (18.2)	*0.395*	69 (2.8) 1024 (42.5) 789 (32.8) 525 (21.8)	0.571	18 (3.1) 296 (50.4) 179 (30.5) 94 (16.0)	0.031 *

**P^a^:***p*-value for comparing between long-term and recent cancer survivors including skin cancer; **P^b^:***p*-value for comparing between long-term and recent cancer survivors excluding skin cancer; **P^c^:***p*-value for comparing all cancer cases (including skin cancer) against the control group (cancer-free population); **P^d^:***p*-value for comparing all cancer cases (excluding skin cancer survivors) against the control group.

**Table 4:** Adherence to individual health behaviours between recent, long-term, and all cancer survivors (with and without skin cancer survivors) compared with controls.

Adherence to Health Behaviours (Coded “1” for Those Who Adhered and “0” for Those Who did not)	Controls *n* = 3896	Cancer Survivors Including Skin Cancer (n = 2407)		Cancer Survivors Excluding Skin Cancer (n = 587)		All Survivors (Long and Recent Survivors) Together	
		Long-Term AOR (95% CI)	Recent AOR (95% CI)	Long-Term AOR (95% CI)	Recent AOR (95% CI)	With Skin Cancer AOR (95% CI)	Excl. Skin Cancer AOR (95% CI)
Body Mass Index (BMI)	Ref.	1.04 (0.87, 1.23)	1.25 (1.02, 1.53) *	0.79 (0.56, 1.11)	1.33 (0.95, 1.85)	1.11 (0.96, 1.29)	1.01 (0.78, 1.29)
Physical activity	Ref.	1.04 (0.89, 1.22)	1.15 (0.94, 1.39)	0.83 (0.62, 1.12)	0.85 (0.61, 1.19)	1.08 (0.94, 1.24)	0.84 (0.67, 1.06)
Smoking	Ref.	1.33 (0.94, 1.89)	1.31 (0.84, 2.04)	1.23 (0.63, 2.42)	0.66 (0.35, 1.24)	1.33 (0.98, 1.79)	0.89 (0.55, 1.44)
Fruit consumption	Ref.	0.94 (0.80, 1.11)	0.80 (0.66, 0.97) *	0.95 (0.70, 1.31)	0.70 (0.50, 0.98)	0.89 (0.78, 1.01)	0.83 (0.65, 1.05)
Vegetable consumption	Ref.	0.96 (0.80, 1.20)	0.92 (0.70, 1.21)	0.80 (0.51, 1.25)	0.82 (0.51, 1.32)	0.94 (0.81, 1.14)	0.80 (0.57, 1.13)
Fruit and vegetable consumption	Ref.	0.98 (0.76,1.31)	0.92 (0.70, 1.24)	0.77 (0.45, 1.31)	0.94 (0.60, 1.56)	0.95 (0.77, 1.22)	0.86 (0.59, 1.25)
Alcohol intake	Ref.	1.05 (0.82, 1.35)	1.26 (0.92, 1.72)	0.97 (0.61, 1.54)	1.27 (0.74, 2.25)	1.12 (0.90, 1.38)	1.08 (0.76, 1.59)
Sugar drinks	Ref.	1.02 (0.84, 1.21)	0.82 (0.65, 1.02)	0.74 (0.52, 1.02)	0.62 (0.42, 0.87)	0.93 (0.78, 1.09)	0.68 (0.53, 0.87) *

Note: * *p*-value less than 0.05. AOR = Adjusted Odds Ratio. All estimates adjusted for age group, reported comorbid chronic diseases, residential area, education status, and occupation status.

**Table 5:** A cancer diagnosis history and other covariates on adherence to multiple behaviours (with and without skin cancer).

Associated Factors	Adherence to Multiple Behaviours ^β^; with Skin Cancer Survivors		Adherence to multiple behaviours ^β^; without Skin Cancer Survivors	
	COR (95% CI)	AOR (95% CI)	COR (95% CI)	AOR (95% CI)
Cancer diagnosis history				
Controls (no cancer history)	1.00	1.00	1.00	1.00
Recent survivors	1.07 (0.93, 1.22)	1.19 (1.04, 1.37) **	0.83 (0.66, 1.05)	0.95 (0.74, 1.21)
Long-term survivors	1.10 (0.98, 1.23)	1.14 (1.02, 1.29) **	0.75 (0.61, 0.93) **	0.80 (0.64, 1.03)
Age group				
63–68 years	1.00	1.00	1.00	1.00
69–73 years	1.08 (0.98, 1.18)	1.16 (1.05, 1.27) *	1.07 (0.93, 1.18)	1.10 (0.98, 1.23)
Residential area/regionality				
Not urban	1.00	1.00	1.00	1.00
Urban	1.21(1.10,1.33) *	1.12 (1.02, 1.24) **	1.21 (1.10, 1.33) *	1.12 (0.99, 1.26)
Current Occupation				
Not paid job	1.00	1.00	1.00	1.00
Paid job	1.16 (1.04, 1.31) *	1.04 (0.93, 1.17)	1.16 (1.04, 1.30)	1.06 (0.92, 1.21)
Education status				
No formal education	1.00	1.00	1.00	1.00
Certificate (intermediate/high school)	1.72 (1.49, 1.99) *	1.49 (1.28, 1.74) **	1.72 (1.49, 1.99) *	1.51 (1.26, 1.80) *
Certificate (diploma/apprenticeship)	2.31 (1.96, 2.71) *	2.05 (1.73, 2.43) *	2.30 (1.96, 2.71) *	2.11 (1.73, 2.58) *
University degree	3.10 (2.61, 3.68) *	2.74 (2.29, 3.28) *	3.10 (2.61, 3.68) *	2.77 (2.24, 3.42) *
Marital status				
Not married	1.00	1.00	1.00	1.00
Married	1.26 (1.14, 1.40) *	1.28 (1.15, 1.42) *	1.26 (1.14, 1.40) *	1.25 (1.11, 1.42) *
Other chronic diseases				
None	1.00	1.00	1.00	1.00
1–2	0.65 (0.58, 0.72) *	0.66 (0.59, 0.73) *	0.65 (0.58, 0.71) *	0.64 (0.57, 0.73) *
≥3	0.34 (0.29, 0.39) *	0.36 (0.30, 0.41) *	0.34 (0.29, 0.39) *	0.36 (0.30, 0.44) *

^β^ Multiple behaviours are grouped into four categories and order as follows for ordinal regression; ≤1 behaviour, 2–3 behaviours, 4 behaviours, and 5–6 behaviours; * *p* < 0.01, ** *p* < 0.05, AOR = Adjusted Odds Ratio, COR = Crude (not adjusted) Odds Ratio. Note: education status was collected at baseline survey of ALSWH in 1996, participants’ age 45–50 years.

## References

[1] WCRF/AICR (2018). Recommendations and Public Health and Policy Implications.

[2] ACS Cancer Treatment & Survivorship Facts & Figures 2016–2017. https://www.cancer.org/research/cancer-facts-statistics/survivor-facts-figures.html.

[3] Demark-Wahnefried W., Rogers L.Q., Alfano C.M., Thomson C.A., Courneya K.S., Meyerhardt J.A., Stout N.L., Kvale E., Ganzer H., Ligibel J.A. (2015). Practical clinical interventions for diet, physical activity, and weight control in cancer survivors. Ca Cancer J. Clin..

[4] Rock C.L., Doyle C., Demark-Wahnefried W., Meyerhardt J., Courneya K.S., Schwartz A.L., Bandera E.V., Hamilton K.K., Grant B., McCullough M. (2012). Nutrition and physical activity guidelines for cancer survivors. Ca Cancer J. Clin..

[5] National Cancer Survivorship Initiative (2013). Living with and beyond Cancer; Taking Action to Improve Outcomes.

[6] Demark-Wahnefried W., Jones L.W. (2008). Promoting a healthy lifestyle among cancer survivors. Hematol. Oncol. Clin. North Am..

[7] Minian N., deRuiter W.K., Lingam M., Corrin T., Dragonetti R., Manson H., Taylor V.H., Zawertailo L., Ebnahmady A., Melamed O.C. (2018). The effects of interventions targeting multiple health behaviors on smoking cessation outcomes: A rapid realist review protocol. Syst. Rev..

[8] Prochaska J.J., Spring B., Nigg C.R. (2008). Multiple health behavior change research: An introduction and overview. Prev. Med..

[9] Jackson S.E., Williams K., Steptoe A., Wardle J. (2014). The impact of a cancer diagnosis on weight change: Findings from prospective, population-based cohorts in the UK and the US. BMC Cancer.

[10] Satia J.A., Campbell M.K., Galanko J.A., James A., Carr C., Sandler R.S. (2004). Longitudinal changes in lifestyle behaviors and health status in colon cancer survivors. Cancer Epidemiol. Biomark. Prev. A.

[11] Sprague B.L., Trentham-Dietz A., Nichols H.B., Hampton J.M., Newcomb P.A. (2010). Change in lifestyle behaviors and medication use after a diagnosis of ductal carcinoma in situ. Breast Cancer Res. Treat..

[12] Steinhilper L., Geyer S., Sperlich S. (2013). Health behavior change among breast cancer patients. Int. J. Public Health.

[13] Hawkins M.L., Buys S.S., Gren L.H., Simonsen S.E., Kirchhoff A.C., Hashibe M. (2017). Do cancer survivors develop healthier lifestyle behaviors than the cancer-free population in the PLCO study?. J. Cancer Surviv. Res. Pract..

[14] Humpel N., Magee C., Jones S.C. (2007). The impact of a cancer diagnosis on the health behaviors of cancer survivors and their family and friends. Support Care Cancer.

[15] Kim M., Choi K.S., Suh M., Jun J.K., Chuck K.W., Park B. (2017). Risky Lifestyle Behaviors Among Gastric Cancer Survivors Compared with Matched Non-cancer Controls: Results from Baseline Result of Community Based Cohort Study. Cancer Res.

[16] Bluethmann S.M., Basen-Engquist K., Vernon S.W., Cox M., Gabriel K.P., Stansberry S.A., Carmack C.L., Blalock J.A., Demark-Wahnefried W. (2015). Grasping the ‘teachable moment’: Time since diagnosis, symptom burden and health behaviors in breast, colorectal and prostate cancer survivors. Psychooncology.

[17] Demark-Wahnefried W., Aziz N.M., Rowland J.H., Pinto B.M. (2005). Riding the crest of the teachable moment: Promoting long-term health after the diagnosis of cancer. J. Clin. Oncol. Off. J. Am. Soc. Clin. Oncol..

[18] Inoue-Choi M., Robien K., Lazovich D. (2013). Adherence to the WCRF/AICR guidelines for cancer prevention is associated with lower mortality among older female cancer survivors. Cancer Epidemiol. Biomark. Prev. A.

[19] Kanera I.M., Bolman C.A., Mesters I., Willems R.A., Beaulen A.A., Lechner L. (2016). Prevalence and correlates of healthy lifestyle behaviors among early cancer survivors. BMC Cancer.

[20] Park B., Kong S.Y., Kim J., Kim Y., Park I.H., Jung S.Y., Lee E.S. (2015). Health Behaviors of Cancer Survivors in Nationwide Cross-Sectional Survey in Korea: Higher Alcohol Drinking, Lower Smoking, and Physical Inactivity Pattern in Survivors with Higher Household Income. Medicine.

[21] Brown W., Bryson L., Byles J., Dobson A., Manderson L., Schofield M., Williams G. (1996). Women’s health Australia: Establishment of the Australian longitudinal study on women’s health. J. Women’s Health.

[22] Loxton D., Powers J., Anderson A.E., Townsend N., Harris M.L., Tuckerman R., Pease S., Mishra G., Byles J. (2015). Online and Offline Recruitment of Young Women for a Longitudinal Health Survey: Findings From the Australian Longitudinal Study on Women’s Health 1989–95 Cohort. J. Med. Internet Res..

[23] Stavrou E., Vajdic C.M., Loxton D., Pearson S.A. (2011). The validity of self-reported cancer diagnoses and factors associated with accurate reporting in a cohort of older Australian women. Cancer Epidemiol..

[24] Heesch K.C., Hill R.L., van Uffelen J.G.Z., Brown W.J. (2011). Are Active Australia physical activity questions valid for older adults?. J. Sci. Med. Sport.

[25] Ainsworth B.E., Haskell W.L., Herrmann S.D., Meckes N., Bassett D.R., Tudor-Locke C., Greer J.L., Vezina J., Whitt-Glover M.C., Leon A.S. (2011). Compendium of Physical Activities: A second update of codes and MET values. Med. Sci. Sports Exerc..

[26] Bruno E., Gargano G., Villarini A., Traina A., Johansson H., Mano M.P., Santucci De Magistris M., Simeoni M., Consolaro E., Mercandino A. (2016). Adherence to WCRF/AICR cancer prevention recommendations and metabolic syndrome in breast cancer patients. Int. J. Cancer.

[27] Song F., Qureshi A.A., Giovannucci E.L., Fuchs C.S., Chen W.Y., Stampfer M.J., Han J. (2013). Risk of a second primary cancer after non-melanoma skin cancer in white men and women: A prospective cohort study. PLoS Med..

[28] Eakin E.G., Youlden D.R., Baade P.D., Lawler S.P., Reeves M.M., Heyworth J.S., Fritschi L. (2007). Health behaviors of cancer survivors: Data from an Australian population-based survey. Cancer Causes Control.

[29] LeMasters T.J., Madhavan S.S., Sambamoorthi U., Kurian S. (2014). Health behaviors among breast, prostate, and colorectal cancer survivors: A US population-based case-control study, with comparisons by cancer type and gender. J. Cancer Surviv. Res. Pract..

[30] Ollberding N.J., Maskarinec G., Wilkens L.R., Henderson B.E., Kolonel L.N. (2011). Comparison of modifiable health behaviours between persons with and without cancer: The Multiethnic Cohort. Public Health Nutr..

[31] James E., Freund M., Booth A., Duncan M.J., Johnson N., Short C.E., Wolfenden L., Stacey F.G., Kay-Lambkin F., Vandelanotte C. (2016). Comparative efficacy of simultaneous versus sequential multiple health behavior change interventions among adults: A systematic review of randomised trials. Prev. Med..

[32] Berdan C.A., Tangney C.C., Scala C., Stolley M. (2014). Childhood cancer survivors and adherence to the American Cancer Society Guidelines on Nutrition and Physical Activity. J. Cancer Surviv. Res. Pract..

[33] Inoue-Choi M., Lazovich D., Prizment A.E., Robien K. (2013). Adherence to the World Cancer Research Fund/American Institute for Cancer Research recommendations for cancer prevention is associated with better health-related quality of life among elderly female cancer survivors. J. Clin. Oncol. Off. J. Am. Soc. Clin. Oncol..

[34] Smith W.A., Li C., Nottage K.A., Mulrooney D.A., Armstrong G.T., Lanctot J.Q., Chemaitilly W., Laver J.H., Srivastava D.K., Robison L.L. (2014). Lifestyle and metabolic syndrome in adult survivors of childhood cancer: A report from the St. Jude Lifetime Cohort Study. Cancer.

[35] Song S., Hwang E., Moon H.-G., Noh D.-Y., Lee J.E. (2015). Adherence to Guidelines for Cancer Survivors and Health-Related Quality of Life among Korean Breast Cancer Survivors. Nutrients.

[36] Spector D.J., Noonan D., Mayer D.K., Benecha H., Zimmerman S., Smith S.K. (2015). Are lifestyle behavioral factors associated with health-related quality of life in long-term survivors of non-Hodgkin lymphoma?. Cancer.

[37] Von Gruenigen V.E., Waggoner S.E., Frasure H.E., Kavanagh M.B., Janata J.W., Rose P.G., Courneya K.S., Lerner E. (2011). Lifestyle challenges in endometrial cancer survivorship. Obstet. Gynecol..

[38] Winkels R.M., van Lee L., Beijer S., Bours M.J., van Duijnhoven F.J., Geelen A., Hoedjes M., Mols F., de Vries J., Weijenberg M.P. (2016). Adherence to the World Cancer Research Fund/American Institute for Cancer Research lifestyle recommendations in colorectal cancer survivors: Results of the PROFILES registry. Cancer Med..

[39] Pinto B.M., Trunzo J.J. (2005). Health behaviors during and after a cancer diagnosis. Cancer.

[40] Lee L., Cheung W.Y., Atkinson E., Krzyzanowska M.K. (2011). Impact of comorbidity on chemotherapy use and outcomes in solid tumors: A systematic review. J. Clin. Oncol. Off. J. Am. Soc. Clin. Oncol..

[41] Drake B.F., Quintiliani L.M., Sapp A.L., Li Y., Harley A.E., Emmons K.M., Sorensen G. (2013). Comparing strategies to assess multiple behavior change in behavioral intervention studies. Transl. Behav. Med..

